# Traumatic brain injury hospital incidence in Brazil: an analysis of the past 10 years

**DOI:** 10.5935/0103-507X.20210036

**Published:** 2021

**Authors:** Randhall Bruce Kreismann Carteri, Ricardo Azevedo da Silva

**Affiliations:** 1 Department of Nutrition, Centro Universitário Metodista - IPA - Porto Alegre (RS), Brazil.; 2 Department of Health and Behavior, Universidade Católica de Pelotas - Pelotas (RS), Brazil.

**Keywords:** Lesões encefálicas traumáticas/epidemiologia, Lesões encefálicas traumáticas/mortalidade, Incidência, Brasil, Brain injuries, traumatic/epidemiology, Brain injuries, traumatic/mortality, Incidence, Brazil

## Abstract

**Objetivo:**

Caracterizar os aspectos demográficos e sociais e o ônus econômico do traumatismo craniencefálico no sistema público de saúde brasileiro na última década.

**Métodos:**

Analisaram-se os dados provenientes da base de dados do Departamento de Informática do Sistema Único de Saúde referentes ao período entre janeiro de 2008 e dezembro de 2019.

**Resultados:**

Entre 2008 e 2019 ocorreram, em média, no Brasil, 131.014,83 internações por traumatismo craniencefálico ao ano, com incidência de 65,54 por 100 mil habitantes. Deve-se salientar a elevada incidência de traumatismo craniencefálico em adultos idosos (acima de 70 anos), acompanhada de altas taxas de mortalidade. Além disso, há também elevada incidência de traumatismo craniencefálico em adultos jovens (20 a 29 anos e 30 a 39 anos). Os dados aqui apresentados demonstram uma proporção de traumatismos craniencefálicos de 3,6 homens/mulheres.

**Conclusão:**

Embora se acredite que os dados apresentados subestimem a incidência e mortalidade associadas com o traumatismo craniencefálico no Brasil, este estudo pode ajudar na implantação de futuras estratégias de promoção da saúde para a população brasileira e mundial, com o objetivo de diminuir a incidência, a mortalidade e os custos do traumatismo craniencefálico.

## Abstract (pt)

**Objective:**

To characterize the demographic, social, and economic burden of traumatic brain injury on the public health system in Brazil during the past decade.

**Methods:**

Data from the database of the Information Technology Department of the Unified Health System (DATASUS) from January 2008 to December 2019 were analyzed.

**Results:**

There was a mean of 131,014.83 hospital admissions per year due to traumatic brain injury in Brazil from 2008 - 2019. The incidence was 65.54 per 100.000 inhabitants during the same period. The high incidence of traumatic brain injury in older adults (>70 years old) accompanied by high mortality rates should be noted. In addition, there was a high incidence of traumatic brain injury in younger adults (20 to 29 years and 30 to 39 years). The data presented here demonstrates a 3.6 male-to-female ratio of traumatic brain injury incidence.

**Conclusion:**

Although we believe that the present data underestimate the incidence and mortality related to traumatic brain injury in Brazil, this study could assist in implementation of future health promotion strategies in the Brazilian population and worldwide aiming to reduce the incidence, mortality and costs of traumatic brain injury.

## INTRODUCTION

Traumatic brain injury (TBI) commonly results in persistent neurological dysfunction and is currently recognized as a major public health problem.^(1,2)^ TBI is defined as impaired brain function resulting from biomechanical forces (i.e., rapid acceleration or deceleration of the brain; direct impact or air blast due to explosions) or penetration of the skull by an object.^(2,3)^ The associated damage may progress from primary mechanical damage to secondary deleterious effects, including progressive neurodegeneration, which is the main cause of TBI-associated disability and death.^(3)^ Nevertheless, the importance of epidemiologic analysis of TBI is important for providing appropriate information for the promotion of primary prevention strategies (to reduce TBI incidence) and reinforcement of the importance of research on secondary and tertiary prevention (for treatment and rehabilitation following injury).^(4)^

The increase in TBI incidence is a worldwide phenomenon, mainly due to traffic accidents and the growth of the elderly population, which is at risk for falls.^(3)^ In Brazil, prior to 2012, there were an estimated 500 cases per 100,000 inhabitants, resulting in a cost greater than 250 million dollars to and 998,994 hospitalizations in the Brazilian Unified Health System (*Sistema Único de Saúde* - SUS), including an average cost of US$ 239,91 for each hospitalization.^(5)^ However, these data on costs do not include outpatient and rehabilitation clinic costs, or medicines; the cost of home care, caregivers, and transportation; and indirect costs (i.e., days not worked by patients and family).^(5)^ TBI and its consequences are currently an important public health problem in Brazil, necessitating a broader and updated evaluation of available nationwide data to provide insight into future directions for public health policies.

Therefore, we aimed to characterize the demographic, social, and economic burden of TBI in Brazil during the past decade using data provided by the Information Technology Department of the Unified Health System (*Departamento de Informática do Sistema Único de Saúde* - DATASUS).

## METHODS

This population-based study used descriptive statistics to characterize TBI hospitalizations in Brazil from 2008 to 2019. Therefore, Ethics Committee in Research/institutional review board approval was considered unnecessary since all data were obtained from a public domain database that is accessible online.

Analysis of the data available from January 2008 to December 2019 was performed. All information used to analyze TBI hospitalization in Brazil is stored in the database of the DATASUS (available online at http://www2.datasus.gov.br). This database is fed by the “hospital admission authorization (HAA)” system by the public and private health institutions that make up the SUS in Brazil. Based on the International Disease Classification, 10th Revision (IDC-10), the terms “skull and bone fracture” and “intracranial trauma” were selected from the “Tabulation List for Morbidity,” which presents a classification specific to the Brazilian SUS context.^(6)^ We opted to exclude “trauma to the eye and eye socket,” considering these injuries to be less specific to TBI.

Data was selected from DATASUS to obtain data on total hospital admissions and total costs of admissions. The costs were direct costs, as indicated by the hospital and imputed in the database (and therefore did not include indirect costs), and costs in dollars were calculated dividing by 4.5 (dollar value compared to Brazilian Real value in March 2020). These data were further stratified by region of occurrence, year, sex and age group. The incidence (number of new cases in the population per year) was calculated with information on the total resident population and age distribution of the population for each year obtained by the agency responsible for official collection of statistical information in Brazil, i.e., *Instituto Brasileiro de Geografia e Estatística* (IBGE - https://www.ibge.gov.br/).^(7)^ Therefore, it should be noted that patients who died due to TBI and were not hospitalized were not included in the analysis.

Finally, we provided r^2^ values for specific observed trends using linear regression, with a significance level threshold of p < 0.05 and forecasted data using modelers for time series with Statistical Package for the Social Sciences (SPSS), version 25.0 for Windows. Graphics were created with Prism 7.0 software.

## RESULTS

**Figure 1:**
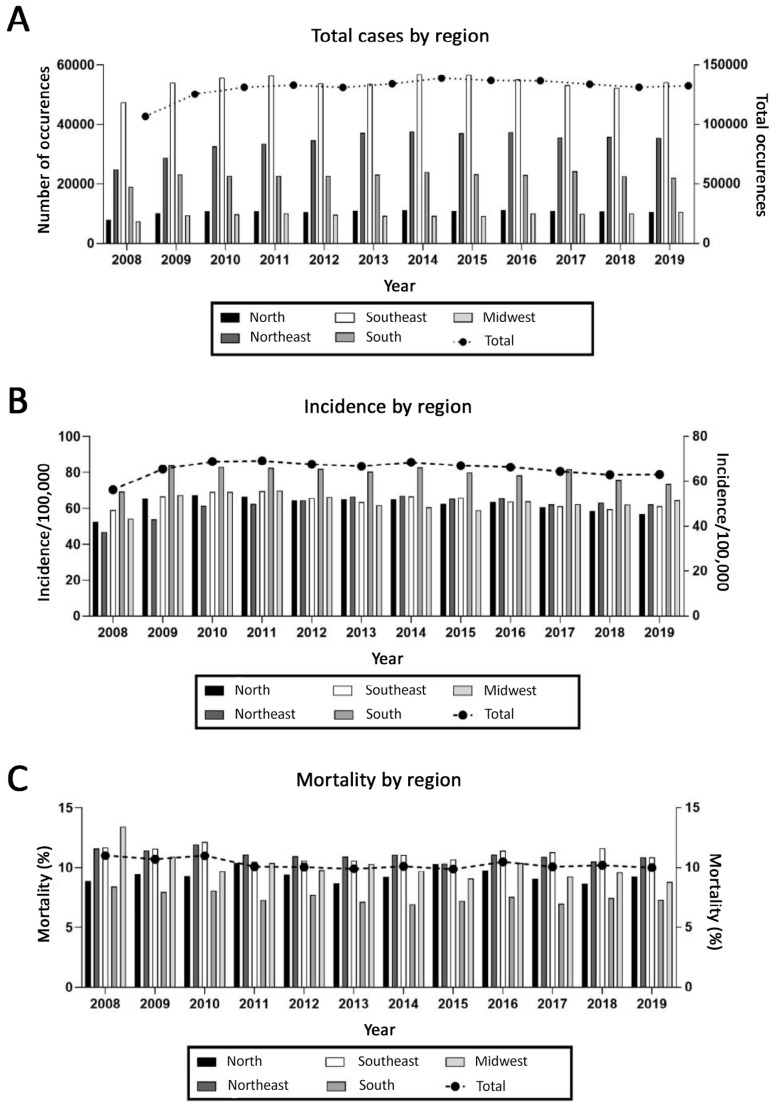
Hospital admissions (A), incidence (B) and mortality (C) due to traumatic brain injury by region and year. The line represents total cases and is plotted on the right Y axis.

Analysis of the available DATASUS data from 2008 - 2019 showed that the total number of hospital admissions was 1,572,178.00. There was a mean of 131,014.83 hospital admissions per year associated with TBI from 2008 - 2019 in Brazil (Table 1). In absolute numbers, the region with the highest admissions was the Southeast (648,447.00), followed by the Northeast and South (410,478.00 and 272,944.00, respectively). The North and Midwest had fewer admissions (126,327.00 and 113,982.00). Figure 1A shows total hospital admissions for each year. The mean annual incidence of admissions was 65.54 per 100,000 inhabitants during the period. A higher mean incidence was observed in the South (79.43), followed by the Southeast, Midwest and North (64.35, 63.41 and 62.37, respectively). The mean incidence was the lowest in the Northeast (61.75). The incidence for each region by year is shown in figure 1B. The mean annual mortality rate associated with TBI in Brazil from 2008−2019 (Table 1) was 10.27. The Southeast and Northeast had the highest mortality rates (11.16 and 11.02, respectively), followed by the Midwest (10.05) and North (9.33), while the South had the lowest rate (7.49). Figure 1C shows mortality rates by year in the different regions.

**Table 1:** Admissions, costs and hospital days

	Mean incidence/100,000	Total admissions	Mean admissions/year	MHS (days)	Mortalidade média
North	62.37	126,327	10,527.25	6.17	9.33
Northeast	61.75	410,478	34,206.50	5.93	11.02
Southeast	64.35	648,447	54,037.25	5.94	11.16
South	79.43	272,944	22,745.33	4.79	7.49
Midwest	63.41	113,982	9,498.50	5.70	10.05
Brazil	65.54	1,572,178	131,014.83	5.74	10.27

MHS - mean length of hospital stay.

The average annual overall cost of hospital expenses associated with TBI patients was approximately US$43,238,319.90, with an average cost per admission of US$327.68. Data regarding the costs due to TBI showed an increasing trend from 2008 to 2019 for all evaluated parameters (Table 2). In addition, forecasts for 2020 are shown in table 2.

**Table 2:** Total costs due to traumatic brain injury by year

	Annual cost	Hospital services	Professional services	MHS(days)
2008	US$23,461,278.67	US$18,987,806.48	US$4,473,472.19	US$219.89
2009	US$32,589,714.37	US$26,595,666.20	US$5,994,048.17	US$259.73
2010	US$36,613,006.67	US$29,837,453.27	US$6,775,553.40	US$278.80
2011	US$39,656,171.41	US$31,911,264.66	US$7,744,906.75	US$297.98
2012	US$41,345,757.49	US$32,974,095.14	US$8,370,611.35	US$315.29
2013	US$44,161,765.56	US$35,348,508.62	US$8,804,784.44	US$329.12
2014	US$46,984,587.22	US$37,732,162.41	US$9,247,906.67	US$338.37
2015	US$48,259,659.33	US$38,833,216.73	US$9,421,491.43	US$352.29
2016	US$50,756,333.31	US$40,764,986.99	US$9,986,079.84	US$371.19
2017	US$51,210,179.86	US$41,097,753.31	US$10,101,723.46	US$382.76
2018	US$51,042,711.36	US$40,966,507.98	US$10,075,868.56	US$388.60
2019	US$52,778,673.56	US$42,491,999.05	US$10,286,219.03	US$398.14
Forecasts				
r^2^	0.952	0.944	0.912	0.982
2020	US$54,514,625.99	US$44,017,475.53	US$10,365,546.95	US$407.67
LCL	US$50,203,929.53	US$40,321,317.49	US$9,586,423.34	US$391.22
UCL	US$58,825,322.46	US$47,713,633.57	US$11,144,670.56	US$424.12

MHS - mean hospital stay; LCL - lower control limit; UCL - upper control limit.

In absolute numbers, higher hospital admissions were observed among older adults (above 70 years old), followed by younger adults (20 to 29 years and 30 to 39 years) (Table 3). In addition, older adults also had higher mortality rates, which increased with age (r^2^ = 0.93) (Table 3).

**Table 3:** Total incidence, number of hospital admissions, number of deaths, mortality associated with traumatic brain injury by age group

Age (years)	Admissions	Deaths	Mortality	Incidence/100,000
> 1 - 4	95,839.00	1,516.00	1.58	651.89
5 - 9	61,893.00	907.00	1.47	401.86
10 - 14	59,306.00	1,558.00	2.63	365.76
15 - 19	137,214.00	7,654.00	5.58	808.89
20 - 29	334,893.00	19,360.00	5.78	968.52
30 - 39	262,429.00	16,752.00	6.38	824.38
40 - 49	206,846.00	16,544.00	8	781.22
50 - 59	156,482.00	15,835.00	10.12	763.15
60 - 69	108,317.00	13,663.00	12.61	786.56
70 - 79	84,796.00	12,864.00	15.17	1145.17
80+	64,162.00	12,689.00	19.78	1799.25

**Figure 2:**
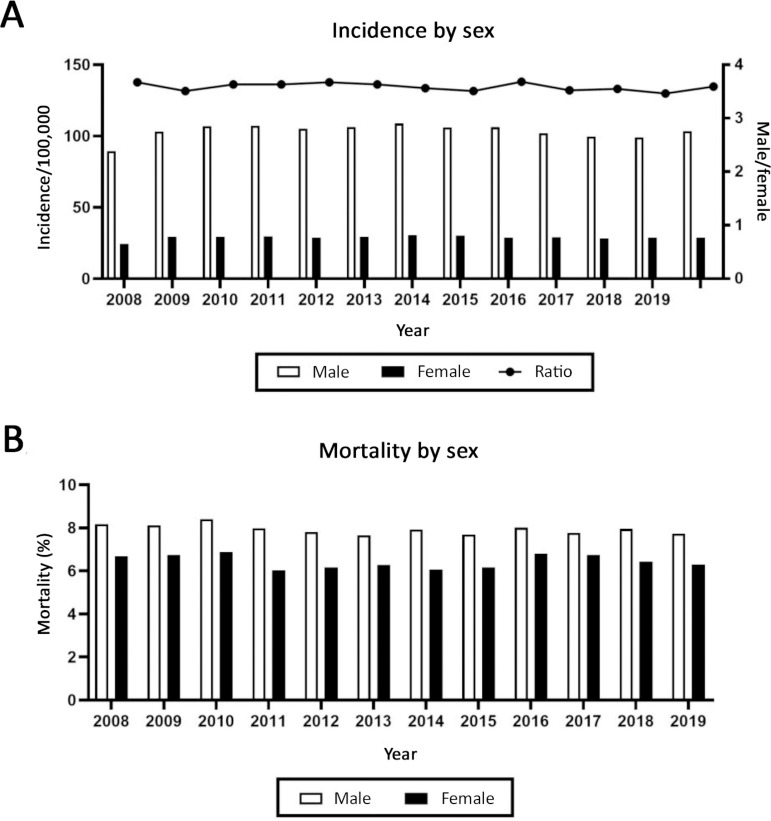
Total annual incidence (A) and mortality (B) by sex.

Furthermore, from 2008 to 2019 in Brazil, incidence and mortality were higher for males than for females. The average incidence was 103.3 for males and 28.83 for females (Figure 2A), and the mean male/female incidence ratio was 3.6. The mean mortality rates were 10.90 for male patients and 8.30 female patients (Figure 2B).

## DISCUSSION

This study aimed to evaluate nationwide epidemiologic data for TBI in Brazil during the past decade. The DATASUS database is contains data from hospitalization forms from the Brazilian public health system (SUS). According to recent data from the National Health Survey in Brazil, the majority of the population (estimated at 80%) is dependent on SUS for medical care.^(8)^ Moreover, SUS also provides care for users of private health plans and private services when they need highly complex care, such as transplants, hemodialysis and high-cost medicines.^(9)^ Therefore, since SUS provides most health care in Brazil, this database is a reliable data source for estimates of TBI-related data for the country.

In Brazil, although there have been several campaigns and public policies warning about the risks of speeding and alcohol abuse associated with driving, currently, one in six emergency room admissions are due to TBI, and most are associated with road traffic collisions; the number of deaths resulting from TBI is only surpassed by that resulting from cancer and cardiovascular disease.^(10,11)^ Other causes of TBI include falls, contact sports, violence, suicide, and objects falling onto the skull.^(12,13)^

There was a mean of 131,014.83 hospital admissions per year due to TBI from 2008 - 2019 in Brazil. The incidence was 65.54 per 100,000 inhabitants during the same period. Compared to the results of the last study using the same database to evaluate data from 2008 to 2012, there was no change in incidence, although there was an increase in the number of annual mean hospital admissions and mortality rates.^(14)^ Considering global estimates of 200 cases per 100,000 inhabitants, the incidence of TBI reported here may be underestimated. Compared to a recent robust regional estimate, Brazil has approximately one-tenth as many cases as is estimated in North America, Europe and African regions; the same study estimated an incidence of 909 for the Latin American region.^(15)^

Current data indicate that the southern region of Brazil has the highest incidence, followed by the Southeast, Midwest and North. Surprisingly, mortality in the South was lower than that in the other regions, and both the Southeast and Northeast showed higher mortality rates. Recently, a multicenter study of TBI in Santa Catarina (a state in the South region of Brazil) reported 9.5 cases per 100,000 habitants per year and a mortality rate of 5.43 per 100,000 habitants per year; the authors speculated that mortality could be underestimated.^(16)^ Differences in urbanization, quality of roads, and access to and quality of health-care services, particularly in rural areas, could explain the discrepancy among regions.^(17)^ Notably, the urgency of the need for better primary prevention strategies is also evidenced by the increase in annual overall costs of TBI in Brazil, which since 2010 has shown an average annual increase of 5%.

The high incidence of TBI in older adults (above 70 years old) accompanied by high mortality rates is of great concern. In addition, TBI incidence was also elevated among younger adults (20 to 29 years and 30 to 39 years), showing that in the Brazilian population, similar peaks in TBI cases occurs in these age groups.^(18,19)^ This was also observed in a previous study of the same population,^(14)^ demonstrating that these age groups remain at high risk for TBI. According to IBGE population projections released in 2018, the population of older Brazilians is growing: the number of older people will exceed that of young people in 2031, when there will be 42.3 million young people (0 - 14 years old) and 43.3 million older adults aged 60 years and over.^(7)^ Falls cause more than one-third of TBI cases within the general population and more than 60% of all TBIs among people older than 65 years, who have the highest rates of both TBI-related hospitalization and death.^(20)^ Thus, future prevention strategies could specifically target these age groups.

Epidemiological studies have indicated that men, especially in the 20- to 29- and 40- to 49-year age groups, are the most susceptible to TBI due to increased risky behavior, with the main cause being associated with traffic accidents.^(21-24)^ The data presented here demonstrate a 3.6 male-to-female ratio for TBI incidence. This pattern has also been observed in other studies.^(24,25)^

Notably, although abundant preclinical evidence has identified emerging potential therapeutic targets against TBI pathophysiology to improve survival, there is currently no available pharmacological intervention with proven efficacy.^(26-28)^ Thus, the observed constant mortality rates over the years are clear evidence that we are still unable to translate preclinical findings to improve outcomes associated with TBI, which remains a challenge.^(29,30)^

Some limitations of this study should be addressed. The research tool for the DATASUS database provides no information on causes of admissions for TBI or TBI severity in Brazil. In Brazil, traffic accidents are the main cause of TBI.^(22,23,31)^ The mortality rate related to traffic accidents decreased from 1990 to 2015, with higher mortality rates in states in the North and Northeastern than in those in the South and Southeast.^(24)^ Considering that we found the highest incidence of TBI in the South as well as a lower mortality rate, this could indicate that the incidence reported here could be underestimated; the data represent hospital admissions, and there are no reports on deaths without hospital admissions.

Although there have been several campaigns and public policies warning about the risks of speeding and alcohol abuse associated with driving, currently, one in six emergency room admissions is due to TBI, and most of them are associated with traffic accidents; the number of deaths resulting from TBI is only surpassed by that resulting from cancer and cardiovascular disease.^(10,11)^ Other causes of TBI include falls, contact sports, violence, suicide, objects falling onto the skull, for which injuries may be underreported.^(12,13)^ In addition, it should be noted that the DATASUS database provides important information, but its limitations do not allow for further insights regarding costs of treatment and rehabilitation for TBI patients and could also result in underestimation of TBI incidence and mortality rates. However, the data discussed here highlight the importance of promoting prevention of this important public health problem and can be useful for future prevention programs.

## CONCLUSION

Epidemiologic vigilance is required to fully understand the impact of traumatic brain injury, a recognized public health problem in Brazil. The high incidence of traumatic brain injury in adults and older populations should also be noted. Although we believe that the present data underestimate traumatic brain injury incidence and mortality in Brazil, this study could assist in the implementation of future health promotion strategies in the Brazilian population and worldwide that aim to reduce the incidence, mortality and costs of traumatic brain injury.
